# Learning curve in robotic surgery for ureteropelvic junction obstruction in children: how to best define a reliable learning process through CUSUM analysis

**DOI:** 10.3389/fsurg.2025.1693109

**Published:** 2026-01-16

**Authors:** Girolamo Mattioli, Maria Stella Cipriani, Maria Grazia Calevo, Martina Monti, Venusia Fiorenza, Marcello Carlucci

**Affiliations:** 1 Pediatric Surgery Department, IRCCS, Istituto Giannina Gaslini, Genoa, Italy; 2 DINOGMI, University of Genoa, Genoa, Italy; 3 Pediatric Surgery Department, Pugliese-Ciaccio Hospital, Catanzaro, Italy; 4 Epidemiology, Biostatistics Unit, IRCCS, Istituto Giannina Gaslini, Genoa, Italy

**Keywords:** children, learning curve, pyeloplasty, robotic surgery, ureteropelvic junction obstruction

## Abstract

**Introduction:**

Robot-assisted laparoscopic pyeloplasty (RALP) is the most common robotic procedure performed in children. The aim was to evaluate the LC of a pediatric urologist experienced in open and laparoscopic surgery, using CUSUM analysis of both operative times and outcomes, to provide a more comprehensive understanding of the learning process.

**Methods:**

This single-centre prospective study included children who underwent RALP for ureteropelvic junction obstruction (UPJO) between February 2021 and October 2024. Demographic, operative and postoperative data were collected. The parameters assessed through CUSUM analysis were total operative time (TOT), plotted using three different reference means; console operative time (COT); and a composite parameter (CP) combining TOT, urological complications, and success.

**Results:**

Twenty-two patients were included. Median age at surgery was 2.1 years, and mean weight 17.1 kg. Mean TOT was 158.1 min, and mean COT was 109.2 min. Median follow-up was 8.8 months. CUSUM-TOT and CUSUM-COT curves, based on the operating surgeon's mean values, identified three phases: introductory (4 cases), proficiency (6 cases), and mastery (12 cases). In contrast, the CUSUM-CP curve revealed only two phases: an introductory phase of 17 patients and a proficiency phase of 5.

**Discussion:**

This study highlights how CUSUM LC analysis in RALP varies depending on both the parameter evaluated and the reference value used. Composite metrics revealed a longer LC than operative time alone, highlighting the influence of postoperative outcomes in assessing surgical competency. These findings emphasize the need for standardized, multidimensional LC assessments in pediatric RALP to better capture the complexity of the learning process.

## Introduction

Ureteropelvic junction obstruction (UPJO) is defined as an impairment in urine flow from the renal pelvis to the proximal ureter, leading to dilation of the collecting system and potential kidney damage. It has an overall incidence of 1 in 1,500 live births and is more common in males, with a 2:1 male-to-female ratio (1). UPJO is most commonly detected through antenatal ultrasound screening.

Over the past fifteen years, robotic-assisted laparoscopic pyeloplasty (RALP) has become increasingly widespread and, where available, is now the preferred surgical approach for treating UPJO in children. An exception is made for smaller children, for whom open pyeloplasty remains favorable due to shorter anesthesia duration and fewer spatial constraints for robotic instruments (2, 3). However, in experienced hands, a minimally invasive approach can also be performed safely (4, 5). The gold standard technique continues to be the Anderson-Hynes dismembered pyeloplasty, first described in 1949 (6).

The superiority of RALP over laparoscopic pyeloplasty (LP) is widely recognized among experts, largely due to its technical advantages. A recent meta-analysis have further confirmed that RALP offers several benefits in the pediatric population. Specifically, RALP has been associated with lower rates of surgical failure, shorter operative times, and fewer postoperative complications, while also promoting faster recovery and shorter hospital stays (7).

The learning curve (LC) of robotic surgery (RS) has been extensively studied. While widely adopted in adult surgery, RS use in children is still evolving, with UPJO being the most common indication. Consequently, the first pediatric LC studies have focused on RALP (8). In 2022 a systematic review reported the fifteen most relevant LC studies on RALP available at that moment, with only four utilizing the cumulative sum (CUSUM) method (9).

Comparative analyses of the LC for RALP and LP have shown that RS allows for more rapid acquisition of technical proficiency. Moreover, skills acquired in one modality appear transferable to the other, suggesting mutual benefit between experiences in RALP and LP (10, 11).

To date, six studies have reported on the LC of RALP using CUSUM analysis (10, 12–16), with all except Kassite et al. (14) focusing on operative times.

The aim of this prospective study was to evaluate the LC of RALP performed by a pediatric urologist, already proficient in open and conventional minimally invasive surgery (MIS), on his initial robotic procedures, to better understand and evaluate the learning process. We employed a rigorous methodology utilizing the CUSUM method (17) to evaluate both operative times and surgical outcomes, with a particular focus on success and complication rates.

## Material and methods

All children (aged 0–18 years) diagnosed with UPJO who consecutively underwent RALP by the study-designated surgeon between February 2021 and October 2024, were prospectively enrolled. The study was approved by the Regional Ethical Committee (reference number 579/2020-DB id 11005), and the informed consent was obtained by all parents prior to surgery.

Exclusion criteria included the presence of major comorbidities, a history of prior surgery for UPJO, or loss to follow-up.

The recorded data included patient demographics, clinical details and diagnostic workup. As part of the preoperative evaluation, all patients underwent preoperative renal ultrasonography (US) and either MAG3 renal scan or functional magnetic resonance urography (fMRU) to evaluate UPJO and confirm surgical indication. fMRU provides information on split renal function, contrast accumulation patterns during the urographic phase, and detailed renal anatomy, and its functional data have been shown to be comparable to those obtained from MAG3 scintigraphy (18).

Data regarding intraoperative times, surgical details, intraoperative complications and need for conversion were collected.

Operative times were meticulously recorded and categorized into four intervals:

- Total Operative Time (TOT)—from skin incision to skin closure,
- Trocar Placement and Docking Time—from skin incision to the start of console use
- Console Operative Time (COT)—duration of the surgeon's active operation at the robotic console
- Dedocking and Skin Closure Time—from the end of console use to skin closure.

Postoperative data included: length of hospital stay, duration of bladder catheterization and peri-renal drainage and postoperative complications graded more than 3a, according to Clavien-Madadi classification (19).

Follow-up included renal US prior to stent removal and, again, within one to three months after its removal. Subsequent US evaluations were performed at 6 months, 12 months, and annually thereafter. If the anteroposterior diameter (APD) increased compared to baseline, an fMRU was performed to assess for UPJO recurrence. Surgical success was defined as the resolution of UPJO at last follow-up, evidenced by either improvement in hydronephrosis on US or a favorable urographic phase on follow-up fMRU, without the need for redo pyeloplasty.

## Surgery

The operating surgeon was a paediatric urologist with substantial experience in both open and conventional MIS and had undergone structured training in dry and wet laboratory environments. A senior consultant provided supervision and proctoring during the initial procedures to ensure adherence to surgical standards and patient safety.

All procedures were performed using the da Vinci Xi® Surgical System (Intuitive Surgical Inc., Sunnyvale, CA, USA). A transperitoneal dismembered pyeloplasty was carried out in all enrolled patients. The patient was placed in a 45° lateral decubitus position with the affected side elevated. Depending on patient size, three or four 8 mm robotic trocars were used. Pneumoperitoneum was established and maintained at a pressure of 10 mmHg. An Anderson-Hynes dismembered pyeloplasty was performed and a ureteral stent placed in all cases.

## Statistical analysis

Descriptive data are presented as means with standard deviation or medians for continuous variables, and as absolute numbers with corresponding percentages for categorical variables.

The LC was evaluated using the CUSUM method (17). CUSUM analysis of a parameter varying during a learning phase is defined as:

$$\mathrm{CUSUM}=\sum_{n=1}^{\infty }\left(\mathrm{Xi}\;-\mu \right)$$

where Xi represents the individual patient's value for a given parameter, and μ denotes the reference mean.

This reference mean may be calculated from the study population or derived from values reported in the literature (9).

The parameters assessed using CUSUM charts included TOT and COT. To illustrate the sensitivity of CUSUM analysis to the choice of mean, CUSUM-TOT was plotted using three different reference means: the mean TOT of the study cohort and two alternative means obtained from published studies on RALP (20, 21). For the CUSUM-COT chart, only the cohort's mean COT was used, due to the absence of published meta-analytical data on this specific parameter.

Subsequently, we sought to develop a composite parameter (CP) that would reflect not only TOT, but also major urological complications and surgical failure, in order to observe how these additional clinical outcomes might influence the LC.

Major urological complications were classified as those with a Clavien-Madadi grade of ≥3a.

As shown in Table 1, the CP was calculated using the following formula:

CP=TOT×SF×CF

**Table 1:** Composite parameter formula and definitions of its components.

Term	Definition/formula	Values/criteria
CP (Composite Parameter)	CP = TOT × SF × CF	TOT = total operative time
SF (Success Factor)	Reflects RALP success in resolving UPJO	1 = no redo pyeloplasty AND either improvement of hydronephrosis on US or favorable urographic phase on follow-up fMRU
		0 = redo pyeloplasty required
CF (Complication Factor)	Adjusts for postoperative complications	1 = no major urological complications
		1.3 = ureteral stenting required due to leakage or sub-stenosis
		1.6 = reoperation required due to anastomotic dehiscence or stenosis

RALP, robotic-assisted laparoscopic pyeloplasty; UPJO, ureteropelvic junction obstruction; US, renal ultrasonography; fMRU, functional magnetic resonance urography.

The Success Factor (SF) was assigned a value of 1 in cases without the need for redo pyeloplasty and with either improvement of hydronephrosis at US or a favorable urographic phase on follow-up fMRU. An SF of 0 was assigned when redo pyeloplasty was required. CF (Complication Factor) was defined as follows: a value of 1 in the absence of major urological complications; 1.3 if the patient required only ureteral stenting due to leakage or sub-stenosis; and 1.6 if reoperation was necessary due to anastomotic dehiscence or stenosis. While both latter scenarios correspond to Clavien-Madadi grade 3a complications, we considered it clinically meaningful to differentiate between them.

CUSUM charts were employed to assess whether distinct learning phases could be identified and characterised. The number of patients of the phases identified in the CUSUM-TOT and CUSUM-CP charts were compared using the Chi-square test. Additionally, patients' demographic, preoperative, and postoperative data across the three phases identified by CUSUM-TOT and CUSUM-COT, as well as the two phases identified by CUSUM-CP, were compared using the Chi-square test. A *p*-value of less than 0.05 was considered statistically significant. Statistical analysis was performed using SPSS Statistical Package for the Social Sciences for Windows version 29 (SPSS Inc. Chicago, IL USA).

## Results

A total of 24 patients met the inclusion criteria. Of these, one was excluded due to lack of follow-up, and another was excluded because of an anatomical variation (specifically, a back-rotated renal pelvis) which led to a prolonged operative time and a technically challenging RALP procedure.

Prenatal diagnosis of UPJO was reported in 72% of cases. The median age at the time of surgery was 2.1 years, with a mean weight of 17.1 kg. The youngest patient was 4.8 months old and weighed 6 kg.

Operative times analysis showed a mean TOT of 158.1 min, and a mean COT of 109.2 min. In all procedures, a ureteral JJ stent or external splint was placed intraoperatively. No conversions to open surgery occurred, and the median postoperative hospital stay was 2 days.

Median follow-up was 8.8 months. Postoperative complications of Clavien-Madadi grade ≥ 3a occurred in 5 patients. One patient developed segmental ileal necrosis due to evisceration through the drain site, requiring ileal resection. The first patient enrolled in the study required a redo open pyeloplasty due to anastomotic dehiscence, while another patient underwent redo RALP for recurrent obstruction. In two cases, ureteral stents were reinserted due to anastomotic leakage following non-functioning external Bracci ureteral splint. All patients underwent renal US both pre- and postoperatively. A reduction in APD of the renal pelvis was observed in every case at the last follow-up. The mean APD decreased from 27.7 ± 12.8 mm before surgery to 8.7 ± 6.9 mm after surgery.

Demographic and operative data are summarized in Table 2.

**Table 2:** Descriptive analysis of study patients.

Preoperative, operative, and postoperative data	Patients [*N* = 22]	
**Demographic and clinical variables before surgery**		
Age at diagnosis (*years*), *median (min-max)*	0 (0–12)	
Age at surgery (*years*), *median (min-max)*	2.1 (0.4–12.6)	
Prenatal diagnosis, *N* (%)	16 (72.3%)	
Gender		
Male, *N* (%)	14 (63.6%)	
Female, *N* (%)	8 (36.4%)	
Weight *(kg), mean ± SD*	17.1 ± 11.8 (6–43)	
Laterality, right/left, *N* (%)	7 (31.8%)/15 (68.2%)	
Crossing vessel, *N* (%)	9 (40.9%)	
Pain, *N* (%)	5 (22.7%)	
UTI, *N* (%)	5 (22.7%)	
Arterial hypertension, *N* (%)	1 (4.5%)	
CKD, *N* (%)	0	
**Intraoperative data**		
Console operative time *(minutes), mean ± SD*	109.2 ± 21.1 (80–155)	
Total operative time *(minutes), mean ± SD*	158.1 ± 22.5 (119–210)	
Trocar Placement and Docking Time *(minutes), mean ± SD*	24.6 ± 8.2 (10–45)	
Dedocking and Skin Closure Time *(minutes), mean ± SD*	24.2 ± 9.3 (9–48)	
Ureteral stent, *N* (%)	22 (100%)	
Drain, *N* (%)	6 (27.3%)	
Conversion, *N* (%)	0	
Intraoperative complications ≥ 3a, *N* (%)	1 (4.5%)	
**Postoperative data**		
Length of hospitalisation *(days), mean ± SD*	2 (0–19)	
Catheter *(days), mean ± SD*	1 (0–17)	
Drain *(days), mean ± SD*	0 (0–14)	
Stent *(days), mean ± SD*	48 (2–161)	
Postoperative complications ≥ 3a		
Overall, *N* (%)	5 (22.7%)	
Urological, *N* (%)	4 (18.2%)	
Re-stentig for leak/stenosis, *N* (%)	2 (9.1%)	
RALP failure (Redo RALP), *N* (%)	2 (9.1%)	
Length of follow-up (months), *median (min-max)*	8.8 (1.7–29.9)	
**Imaging data before and after surgery**	Pre-operative	Post-operative
US APD *(mm), mean ± SD*	27.7 ± 12.8 (15–79) [*n* = 22]	8.7 ± 6.9 (0–30) [*n* = 22]
fMRU APD *(mm), mean ± SD*	30.6 ± 15.0 (15–83) [*n* = 18]	22 ± 13.2 (12–45) [*n* = 5]
Split function of the affected kidney at fMRU *(%), mean ± SD*	33.4 ± 9.6 (18–51) [*n* = 17]	40.0 ± 7.8 (28–48) [*n* = 5]
Split function of the affected kidney at MAG3 renal scan *(%), mean ± SD*	22.9 ± 27.8 (11–93) [*n* = 11]	- [*n* = 0]

UTI, urinary tract infection; CKD, chronic kidney disease; RALP, robotic-assisted laparoscopic pyeloplasty; US, renal ultrasonography; fMRU, functional magnetic resonance urography; APD, anteroposterior diameter.

## Learning curve analysis

**Figure 1:**
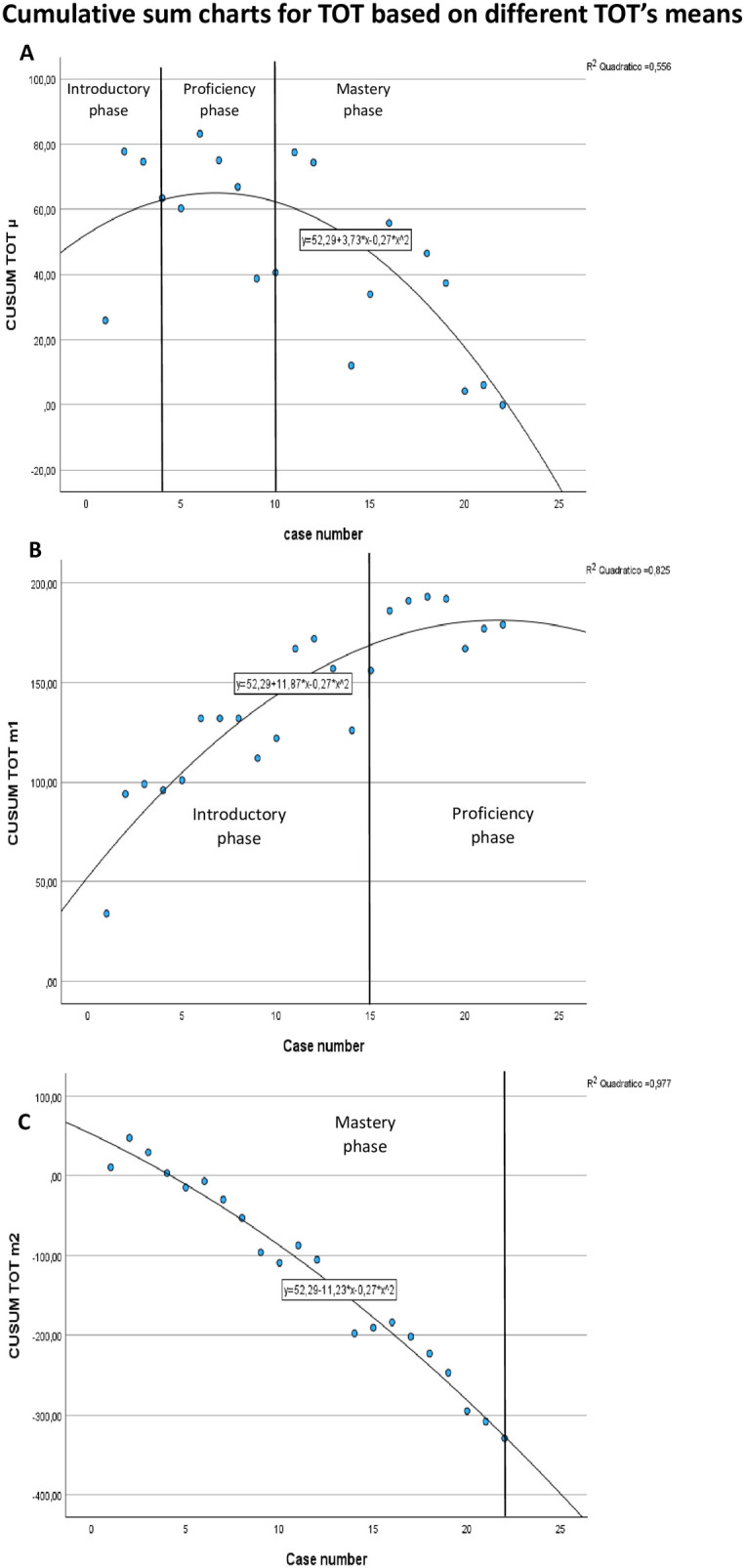
Cumulative sum (CUSUM) charts for total operative time (TOT). **(A)** Based on the mean TOT of the operating surgeon (µ = 109.2 min). **(B)** Based on the mean TOT for RALP reported by Ekin et al. (m1 = 150 min). **(C)** Based on the mean TOT for RALP reported by Silay et al. (m2 = 173.1 min).

A CUSUM analysis was performed to evaluate the learning process of a pediatric urologist during their first RALP procedures. The CUSUM curve for Total Operative Time was performed using three different reference values: the operating surgeon's mean TOT and two benchmark TOTs reported in the literature (20, 21). These three curves are presented in Figure 1.

Chart A illustrates the CUSUM-TOT curve using the surgeon's own mean TOT as the reference, revealing three distinct learning phases:

- Phase 1 (introductory): the first 4 cases
- Phase 2 (proficiency): the following 6 cases, forming a plateau
- Phase 3 (mastery): the final 12 cases, in which the surgeon's performance rapidly improves, consistently surpassing his own average.

In contrast, Chart B, based on the literature-reported mean TOT of 150 min by Ekin et al. (20) demonstrated a longer introductory phase of 15 cases, followed by a 7-case proficiency phase, without evidence of a mastery phase, indicating a slower progression when evaluated against this external benchmark. Chart C, using the higher reference mean TOT of 173.1 min reported by Silay et al. (21), showed a different pattern altogether, with the surgeon's operative times remaining below the reference threshold from the outset, suggestive of a high level of performance from the beginning and thus displaying only a mastery phase.

**Figure 2:**
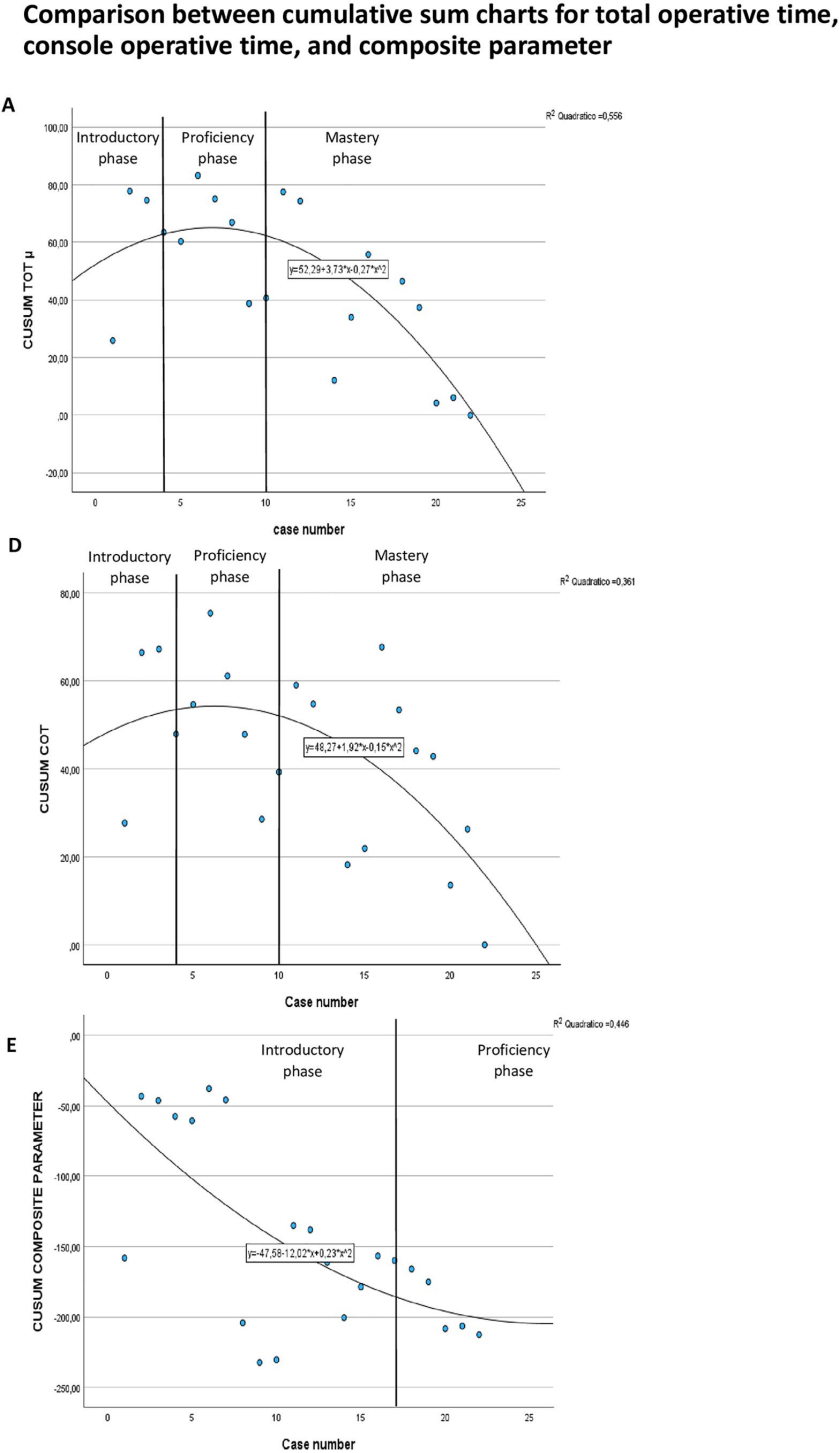
Comparison between cumulative sum (CUSUM) charts for total operative time (Chart **A**), console operative time (Chart **D**), and composite parameter (Chart **E**).

Subsequently, the LC was reassessed using the surgeon's mean Console Operative Time as a reference and compared to the CUSUM-TOT curve shown in Chart A (Figure 2). The resulting CUSUM-COT curve exhibited the same phase distribution, each comprising the same number of patients: an initial introductory phase (4 cases), followed by a proficiency phase (6 cases), and a mastery phase (12 cases).

The data of patients of the 3 phases identified by CUSUM-TOT and CUSUM-COT curves were compared and reported in Table 3. There were no statistically significant differences in age, weight, preoperative imaging, complication rates, or surgical outcomes among the three phases (*p* > 0.05). However, a significant difference was observed between phase 3 and phase 1 in length of hospital stay (*p* = 0.04), presence of a drain (*p* = 0.03), and duration of perirenal drainage (*p* = 0.04). Regarding operative times, no significant differences were found in Trocar Placement and Docking Time or in Dedocking and Skin Closure Time across the three phases (*p* > 0.05). In contrast, both TOT and COT showed statistically significant reductions when comparing phase 2 vs. phase 1 and phase 3 vs. phase 1 (*p* < 0.05).

**Table 3:** Patient demographics, operative details, and postoperative data stratified by phases defined using CUSUM charts based on the mean total operative time (TOT) and mean console operative time (COT) of the operating surgeon.

Comparison of patient data across three learning curve phases	Phase 1	Phase 2	Phase 3	*p value*	*p value*	*p value*
	Introductory	Proficiency	Mastery	1 vs. 2	1 vs. 3	2 vs. 3
	[*n* = 4]	[*n* = 6]	[*n* = 12]			
Age at diagnosis *(years), mean ± SD*	3.00 ± 6.00	4.55 ± 4.67	0.14 ± 0.28	0.73	0.86	0.13
Age at surgery *(years), mean ± SD*	4.27 ± 5.66	6.38 ± 3.63	2.44 ± 3.05	0.61	0.60	**0**.**05**
Age at surgery ≤3 years, *N* (%)	2 (50)	1 (16.7)	10 (83.3)	0.50	0.24	**0**.**01**
Weight *(kg), mean ± SD*	17.50 ± 16.46	23.30 ± 10.20	13.8 ± 10.65	0.47	0.86	0.08
Male, *N* (%)	2 (50.0)	5 (83.3)	7 (58.3)	0.50	1	0.60
Preoperative US APD *(mm), mean ± SD*	25.25 ± 1.70	35.67 ± 22.17	24.58 ± 6.71	0.77	0.95	0.29
Preoperative fMRU APD *(mm), mean ± SD*	30.00 ± 10.74	50.67 ± 28.54	25.41 ± 5.83	0.23	0.85	0.06
Preoperative Split function of the affected kidney at fMRU *(%), mean ± SD*	38.75 ± 8.77	26.33 ± 4.51	33.40 ± 10.22	0.11	0.30	0.37
Console operative time *(minutes), mean ± SD*	137.50 ± 19.77	102.83 ± 16.42	103.08 ± 16.26	**0**.**04**	**0**.**01**	0.89
Total operative time *(minutes), mean ± SD*	182.25 ± 22.51	152.17 ± 16.53	153.08 ± 21.06	**0**.**04**	**0**.**04**	0.68
Trocar Placement and Docking Time *(minutes), mean ± SD*	26.75 ± 13.25	26.33 ± 3.44	23.08 ± 8.33	0.91	0.52	0.08
Dedocking and Skin Closure Time *(minutes), mean ± SD*	18.00 ± 3.56	23.0 ± 9.05	26.92 ± 10.02	0.35	0.06	0.89
Length of hospitalisation *(days), mean ± SD*	9.00 ± 7.16	3.00 ± 1.67	2.50 ± 1.57	0.11	**0**.**04**	0.61
Catheter *(days), mean ± SD*	7.00 ± 7.12	1.17 ± 0.98	1.42 ± 1.16	0.07	0.06	0.68
Drain *(days), mean ± SD*	5.75 ± 6.55	1.00 ± 1.67	0.33 ± 1.15	0.26	**0**.**04**	0.44
Stent *(days), mean ± SD*	33.50 ± 30.38	50.00 ± 39.14	57.67 ± 39.73	1	0.52	0.55
Drain placement, *N* (%)	3 (75)	2 (33.3)	1 (8.3)	0.52	**0**.**03**	0.24
Intraoperative complications ≥ 3a, *N* (%)	0 (0)	0	1 (8.3)	-	1	1
Postoperative complications ≥ 3a						
Overall, *N* (%)	3 (75.0)	1 (16.7)	2 (16.7)	0.19	0.06	1
Urological, *N* (%)	2 (50)	1 (16.7)	1 (8.3)	0.50	0.14	1
Preoperative US APD < Postoperative US APD at last follow-up, *N* (%)	4 (100)	6 (100)	12 (100)	-	-	-
RALP success rate, *N* (%)	3 (75.0)	5 (83.3)	12 (100)	0.47	0.25	0.33

Bold value, statistically significant *p*-value; US, renal ultrasonography; APD, anteroposterior diameter; fMRU, functional magnetic resonance urography; RALP, robotic-assisted laparoscopic pyeloplasty.

Finally, the learning process was also analysed using a composite parameter (CP), and the resulting CUSUM-CP curve was compared to the CUSUM-TOT curve (Chart E vs Chart A in Figure 2). Unlike the previous analyses, the CUSUM-CP curve (Chart E) identified only two learning phases: a prolonged introductory phase involving 17 patients and a shorter proficiency phase of 5 patients. The number of patients in each phase identified in chart E (17, 5, and 0) was significantly different from those in chart A (4, 6, and 12), with a *p*-value < 0.001. Moreover, the data of patients of the two phases identified by CUSUM-CP curve were compared, but no statistically significant differences were found.

## Discussion

Minimal invasive surgery has become a cornerstone of pediatric surgery practice, with RS representing a significant advancement in this field. Since its introduction in 1999 with the Da Vinci system, RS has gained widespread acceptance, particularly in urology. RALP was the first pediatric urologic procedure in which robotic advantages were clearly demonstrated.

In the United States, a 2018 retrospective cohort study reported a decline in open surgery and laparoscopy for UPJO, alongside a steady increase in RALP use, particularly in children and adolescents, with robotic procedures accounting for over 40% of minimally invasive procedures (3). Similarly, a 2020 multicentre study from 26 hospitals identified renal pelvis and ureter surgeries as the most common pediatric urologic procedures performed robotically and those with the greatest growth over time (22).

Nevertheless, open pyeloplasty remains the most common approach in infants, likely due to faster recovery and concerns about hemodynamic and respiratory risks with MIS. Recent evidence, however, supports the safety and feasibility of MIS for UPJO in infants, with meta-analyses showing comparable perioperative outcomes and success rates to open surgery, albeit with longer operative times (3, 23).

Most experts agree on the technical superiority of RALP over LP due to improved ergonomics, high-resolution three-dimensional vision, tremor filtering, and instruments with articulation like that of the human wrist. Moreover, RALP is associated with lower failure rates, shorter operative and anastomotic times, fewer complications, and shorter hospital stays, although it entails substantially higher costs (7, 24).

Given its increasing use in pediatrics, defining adequate training and competency pathways has become a priority in countries with the resources to afford RS.

The LC refers to performance improvement over time. Its application to surgery is necessary for both patient safety and outcomes and healthcare resource optimization (8). RS appears to facilitate a faster LC compared to laparoscopy, with evidence suggesting skill transferability between the two modalities (10, 11). However, LC assessment remains inconsistent due to the lack of standardized definitions, variable outcome measures, and heterogeneous study designs (9).

The aim of this study was to evaluate the LC of RALP performed by a pediatric urologist, at the beginning of their robotic experience, using the CUSUM method, focusing both on operative time and outcomes, to better understand the learning process. The most common method for evaluation of LC consists of simple chronological raw data plots, in contrast CUSUM is a statistical process control tool used to quantitatively assess consecutive performances, which may detect trends that may otherwise be undetected due to natural data variability (12).

To our knowledge, six published studies have reported on the LC of robotic surgeons performing RALP using CUSUM analysis (10, 12–16). All these studies used operative time as the primary learning parameter.

In details, Cundy et al. (12) analysed by CUSUM method a single surgeon's performance in 90 RALP, evaluating setup time, docking time, console time, operating time, and total operating room time. Similarly, Stern et al. (13) examined step specific operative times of 40 RALPs performed by a single surgeon. Andolfi et al. (10) assessed total operative time of a single surgeon performing 39 RALP, comparing it with LC of open and LP of two different surgeons. Xing et al. (16) reported on a large patient cohort operated on by a single surgeon at a single institution, using CUSUM analysis of operative time. Zhou et al. (15) also focused on a single surgeon, evaluating console time and average suture time per stitch as learning indicators. Notably, Kassite et al. also analysed an additional parameter that combined operative time with surgical outcomes, analysing 42 RALP performed by different 2 surgeons without experience in LP (14). Additional studies have evaluated the LC for trainees performing RALP (25, 26).

Comparison of the cited studies' results is challenging because of heterogeneity in clinical settings, surgeons' previous experience, study periods, parameters analysed, and selected targets for CUSUM analysis.

In our series of 22 patients, who underwent RALP performed by the study-designated paediatric urologist between February 2021 and October 2024, demographic and preoperative characteristics were consistent with those reported in the literature. In detail, 72.3% of the children had a prenatal diagnosis, and 63.6% were male. Left-sided UPJO was the most frequent presentation (68.2%). The median hospital stay was 2 days (range 0–19), which is comparable to data reported in the literature. The success rate in our cohort was 90.9%, with a urological complication rate of 18.2%. Although these outcomes are less favorable than those described in previous reports, it should be noted that our data reflect the first 22 RALP procedures performed by a pediatric urologist. In a recent meta-analysis, the reported success rate for RALP is approximately 98.4%, with a complication rate of 3.7% (7).

The surgeon's mean TOT and COT were 158.1 and 109.2 min, respectively. Reported mean TOTs in previous LC studies vary widely, ranging from 142.2 to 232 min (10, 12–14, 16). Notably, only Cundy et al. (12) reported the mean COT during the learning period, which was 167.8 min.

Through CUSUM analysis, we generated five distinct LC charts, the first three, based on TOT, are shown in Figure 1. The distinct shapes of the three curves illustrate how the LC assessed by CUSUM varies, defining different numbers of learning phases and different patient distributions within each phase, depending on the reference mean TOT used.

In our analysis, the CUSUM-TOT curve based on the operating surgeon's mean TOT (Chart A, Figure 1) identified three phases, introductory, proficiency, and mastery, comprising 4, 6, and 12 patients, respectively. These phase distributions differ from those in earlier studies, this discrepancy is likely due to variations in sample size, study duration, and the inherent learning variability between surgeons.

Additionally, when assessing CUSUM curves based on two different literature-derived TOT means (Chart B and C, Figure 1), we observed that the choice of reference value significantly influences the interpretation of learning phases. This finding may explain inconsistencies among published studies and highlights the importance of selecting appropriate reference values, as most use the surgeon's mean operative time while Kassite et al. employed a literature-derived value (14). Nevertheless, establishing a reliable meta-analytical reference TOT for RALP remains difficult due to the lack of multicentre studies that are not affected by confounding factors such as surgeon experience and institutional practices. Based on these considerations, we contend that, to date, the most accurate representation of the LC is achieved using a CUSUM chart with a reference mean derived from the specific study population (e.g., the cohort's own TOT).

Subsequently we analysed the surgeon LC applying CUSUM to COT using the surgeon's mean COT as a reference and compared the resulting chart to the CUSUM-TOT curve based on the operating surgeon's mean TOT (Chart A and D, Figure 2). Prior to the analysis, we hypothesized that COT would be the more appropriate parameter to assess LC, as it more directly reflects the individual surgeon's performance, whereas TOT reflects the performance of the entire surgical team. However, we recognized that COT could still be influenced by factors beyond the console surgeon's control, for example, the time required for stent placement, which depends on the experience level of the bedside assistant. Unexpectedly, Chart D (CUSUM-COT) exhibited a curve similar in shape to Chart A (CUSUM-TOT), with an identical distribution of patients across the three learning phases. Furthermore, neither Trocar Placement and Docking Time nor Dedocking and Skin Closure Time showed a statistically significant difference among the three phases (*p* > 0.05) as shown in Table 3. This may be attributed to these steps being performed by rotating surgical residents at various stages of training throughout the study period, likely minimizing their impact on overall TOT trends.

Then we performed a comparison between patients of the three phases identified by CUSUM-TOT and CUSUM-COT (Table 3). Consistent with previous literature (13, 15, 16), our results showed that both TOT and COT decreased significantly when comparing phase 2 vs. phase 1 and phase 3 vs. phase 1. A significant reduction was also observed in hospital stay between phase 3 and phase 1. Furthermore, the use of perirenal drains and the duration of perirenal drainage decreased significantly in our study, which could be attributed to increased confidence in suturing techniques gained through experience.

The most compelling result is shown in Figure 2. Here, we conducted a CUSUM analysis using a composite parameter influenced by TOT, success of the procedure and major urological complications. While the CUSUM-TOT and CUSUM-COT charts display a triphasic pattern, the CUSUM-CP chart for the 22 enrolled patients is biphasic (Chart E, Figure 2). This composite model modified the LC, increasing the number of cases required to exit the introductory phase from 4 to 17. Moreover, in this LC model, the surgeon has not yet reached the mastery phase. These findings highlight the importance of incorporating multiple performance metrics, not just operative time, into LC analyses. The comparison of patient data between the two phases identified by the CUSUM-CP curve did not show statistically significant differences, which may be attributed to the small sample size and the unequal number of patients in each phase.

In the formula used to calculate the composite parameter, we defined the complication factor by including only major urological complications, unlike Kassite et al. (14) who incorporated all types and grades of complications. This difference could explain discrepancies in the resulting CUSUM analyses.

In summary, our findings suggest that although the surgeon transitioned out of the introductory phase after 4 cases when assessed by TOT alone, a more comprehensive evaluation incorporating SF and CF revealed that 17 cases were required.

These preliminary results suggest that, although the CUSUM analysis of operative time indicates the surgeon has reached the mastery phase, the CUSUM curve for the composite parameter still places the surgeon in the proficiency phase, indicating that further experience is required to achieve full mastery.

We believe that future LC assessments in RALP should always include multiple parameters to provide a more accurate and clinically meaningful representation of surgical proficiency.

## Limitations of the study

The main limitation of this study is the small number of cases. In addition, the composite parameter we used is not validated in the literature, and the weighting of complications is somewhat arbitrary. Furthermore, the minimum follow-up period is relatively short.

## Conclusions

This prospective study highlights how LC analysis in RALP varies depending on both the parameter evaluated and the reference value used in CUSUM analysis.

In our experience, while the surgeon achieved the mastery phase after only 10 cases when assessed based on operative time alone, the CUSUM curve incorporating a composite parameter (including operative time, surgical success, and major urological complications) indicated that the surgeon remained within the proficiency phase even at the 22nd case.

These findings suggest that relying solely on time-based metrics may underestimate the complexity and duration of the actual learning process.

We propose that future LC assessments in RALP should be guided by standardized study protocols, developed through consensus among pediatric urologists, to define the most appropriate and multidimensional parameters. This would allow for a more comprehensive and accurate characterization of the LC in RS for UPJO.

## Data Availability

The raw data supporting the conclusions of this article will be made available by the authors, without undue reservation.
